# Calcium, a multitasking signaling actor in airway smooth muscle cells, as a target of novel strategies to limit airway disease?

**DOI:** 10.3389/fphar.2014.00296

**Published:** 2015-01-14

**Authors:** Stephane Tanguy

**Affiliations:** ^1^Biology, CNRS, TIMC-IMAG Laboratory CNRS UMR 5525, University Joseph Fourier-Grenoble 1, University of GrenobleGrenoble, France

**Keywords:** calcium, smooth muscle cells, airways, signaling pathways, pathophysiology

Asthma, Chronic Obstructive Pulmonary Disease (COPD),… words that have entered our everyday vocabulary, but more especially diseases whose prevalence is growing steadily and which faithfully illustrate the continuous increase in respiratory diseases. The quantified estimations, suggesting that asthma affects 300,000 persons worldwide and that COPD will become the third cause of death in 2020, give a very clear vision of the future of these diseases. Thus, the investigation of the cellular mechanisms of the functioning of the respiratory system has led to the identification of signaling pathways in which modifications could be responsible for the appearance of pathophysiological situations.

The ubiquitous nature of the role of calcium as a signaling actor in smooth muscle cells (SMC) associated with the fact that the SMC are major constituents of the airways, are two basic findings that clearly justify the importance of this book edited by Yong-Xiao Wang. Thus, the union of the 25 articles by almost 50 contributors that make up the 460 pages of this book clearly illustrates the variety and importance of the role of calcium in airway SMC (ASMC). However, more than a simple illustration, this book is a form of “state of the art” of the role of calcium in the physiology of ASMC and then, in its potential involvement in various pathophysiological conditions.

However, it is first necessary to recognize the importance of Yong-Xiao Wang, who has gathered contributors recognized as experts in the field. The numerous works published by Yong-Xiao Wang on this subject therefore confer him the legitimacy to be the editor of such a book based on a simple observation: the number of scientific research is steadily increasing without a “*comprehensive book compiling and detailing the (our) state-of-the-art advances*” (preface p. v) is available. One reason for this increased interest in the role of calcium in ASCM directly comes from the involvement of alterations in regulating the calcium movements in pathophysiological situations. In this context, although the findings presented by Anderson in 1983 in one of the 1st review available on this subject seem very distant, he was clearly establishing the basis for all work presented here: “*It cannot be excluded that changes in one or more of these mechanisms, induced by mediators, hormones, or other agents may be a contribution factor to airway hyperreactivity*” (Anderson, 1983).

Therefore, in the context of recent data collection on this theme, a book review on “*calcium signaling in airway smooth muscle cells*” should probably aim to try to offer an overview of this subject. As mention above, since this book is clearly a state of the art in the field, it seems to me that a figure summarizing the physiological pathways involving calcium in SMC could be the main asset that a short “book review” can provide (Figure 1). It will enable readers to get an overview of the calcium-dependent pathways that are targets to consider in pathophysiological situations.

**Figure 1:**
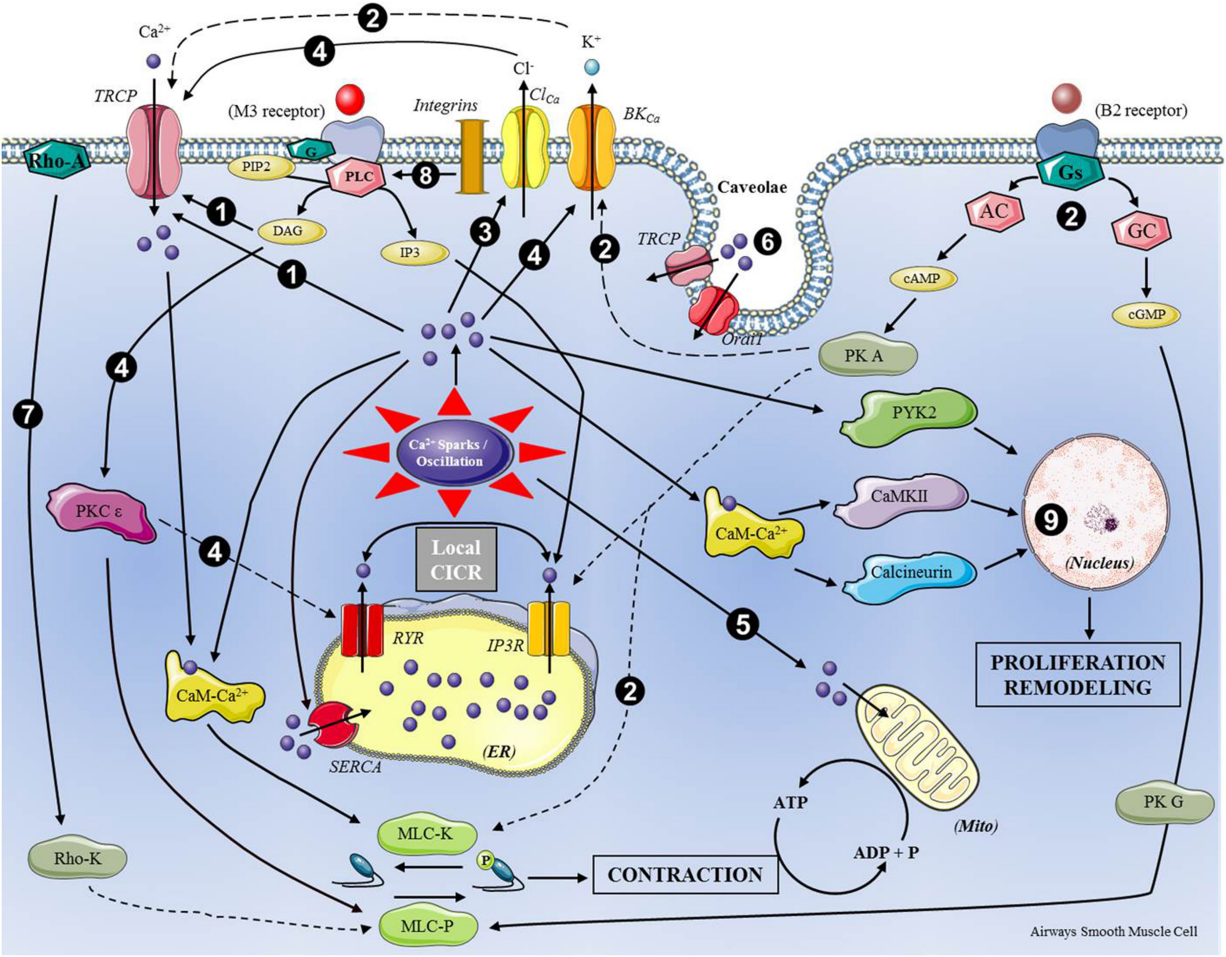
**Calcium-dependent pathways in airways smooth muscle cells**. This figure summarizes the main effects of intracellular calcium dynamics (Ca^2+^-Sparks/Oscillations) from calcium entry into cell and local Calcium-Induced Calcium-Release (CICR) resulting from Endoplasmic Reticulum (ER) protein (RYR, Ryanodine Receptor; IP3R, inositol triphosphate Receptor) activation. The numbers in black circles refer to the following chapters of the book: 1, Mei et al. (pp. 3–20); 2, Kume (pp. 51–83); 3, Gallos et al. (pp. 86–106); 4, Liu et al. (pp. 109–124); 5, Delmotte et al. (pp. 211–234); 6, Pabelick et al. (pp. 235–246); 7, Ito (pp. 287–307); 8, Tran and Teoh (pp. 310–320); 9, Song et al. (pp. 393–407).

Figure 1 shows the intracellular mechanisms involving the calcium in the operation of the ASCM. The main roles presented here show the involvement of calcium in the control of the contraction and relaxation of ASCM but also its role in the control of cell proliferation. Although the main calcium-dependent pathways in ASMC are recalled in Figure 1, they can be modulated by sex hormones or maturation as well-described by Y.S. Prakash et al. in two chapters (pp. 322–332; 333–357). Moreover, since the respiratory pathologies are unanimously recognized as associated with inflammatory situations, the three chapters describing the influence of inflammatory mediators (H. Matsumoto pp. 359–379; Y. Amrani pp. 423–439; Y. Su pp. 441–457) on Ca-dependent pathways in ASMC perfectly complement this book. Finally this synthesis can be well-completed by the two chapters presenting an interesting approach based on mathematical simulation of calcium oscillation in the SMC. Therefore, the mathematical models of calcium dynamics proposed by Sneyd et al. (pp. 341–357) have to be related with the observations of E. Roux (pp. 147–175) on the kinetics of the mechanisms involved in calcium homeodynamics.

In conclusion by drawing an updated synthesis of advanced knowledge of calcium-dependent intracellular pathways in the ASCM, this authoritative book can claim to be an important tool to the use not only for researchers but also for teachers and students.

## Conflict of interest statement

The author declares that the research was conducted in the absence of any commercial or financial relationships that could be construed as a potential conflict of interest.
